# P03 Long term denosumab treatment in osteoporosis - a case series

**DOI:** 10.1093/rap/rkad070.024

**Published:** 2023-09-27

**Authors:** Nimanthi Premathilake, Anupama Nandagudi

**Affiliations:** Department of Rheumatology, Basildon University Hospital, Basildon, United Kingdom; Department of Rheumatology, Basildon University Hospital, Basildon, United Kingdom

## Abstract

**Introduction:**

Studies have shown that denosumab reduces fracture risk and increases bone mineral density and is associated with low adverse events over 10 years. We have reviewed 3 patients in our hospital who have been successfully treated with denosumab 20 injections.

**Case description:**

Clinical details of these patients are outlined in the table.

**Discussion:**

All three patients have responded well to denosumab therapy with definite improvement of T scores and positive bone mineral density change compared to baseline, in both spine and hip regions. They have not experienced significant side effects related to treatment. The second patient was treated as possible fracture as the x-rays were not convincing of definitive fracture. Patient 3, despite having very low T scores and multiple fragility fractures in 2012, has not incurred further fractures following the commencement of denosumab.

**Key learning points:**

Denosumab has shown satisfactory improvement in these patients without significant adverse effects, following completion of 10 years of therapy. This is in par with previous studies that demonstrated efficacy and safety profile of Denosumab.

**Table:** P03 Table

	Patient 1		Patient 2		Patient 3	
Demographic data:						
Age	86 years		78 years		84 years	
Ethnicity	Caucasian		Caucasian		Caucasian	
Gender	Female		Female		Female	
Risk factors for Osteoporosis	Vertebral fractures in 2004 - T6,9,12 L1,3		Premature menopause at 42 years		Low trauma fracture to elbow,wrist and patella in 2012 Hysterectomy at age 48 years	
Treatment received before Denosumab and reasons for discontinuation	Didronel 1 year Risedronate 5-6 years Strontium 2 years - proximal muscle pain Teriparatide 4 months intolerant Total bisphosphonate 12 years		Could not tolerate bisphosphonate and strontium ranelate		HRT for 10 years Raloxifene 3 years Alendronate 6 months Pamidronate 3 years and Ibandronate 4 years (total IV bisphosphonate 7 years) strontium ranelate intolerant	
Date started on Denosumab	2011 September		2011 August		2012 October	
DEXA T-scores before Denosumab	2010 July		2013 September		2012 December	
	Spine	−1.6	Lumbar spine	−2.7	Spine	− 4.6
	NOF	−1.6	NOF	−1.7	NOF	− 3.8
	Hip	−1.3	Hip.	−1.8	Hip	− 3.2
Most recent DEXA T scores	May 2022		2022 August		2023 May	
	Spine	−1.7	Spine	−1.8	Spine	− 3.2
	(L1 & 4 excluded due to degenerative changes)					
	NOF	−0.6	NOF	− 2.3	NOF	−2.8
	Hip	−0.9	Total hip	−1.7	Hip	− 2.7
BMD change Vs Baseline before Denosumab	Spine	−4.3%	Spine	0.6%	Spine	7.5%
	Hip	−1.4%	Hip	−2.8%	Hip	5.8%
BMD change Vs Baseline after 20 injections of Denosumab	Spine	4.7%	Spine	16.3%	Spine	26.5%
	Hip	−0.9%	Hip.	9.8%	Hip	12.9%
Side effects	None		Suspicious line on distal radius – treated as left distal radius possible hairline fracture –2020		None	
20th dose completed on	July 2022		July 2022		April 2023	
Plan	To continue denosumab		To continue denosumab		To continue denosumab	

